# A Case of Menstruation by a Child Five Years of Age

**Published:** 1866-09

**Authors:** A. E. Ames

**Affiliations:** Minneapolis, Minn.


### A case of Menstruation by a Child five years of age.

BY A. E. AMES, M. D., MINNEAPOLIS, MINN.

Addie E. Tinsley, daughter of G. W. Tinsley, of Minneapolis, Minn. Her parents are healthy people. From her birth, until she was four and a half years old, she was never sick. At this time she was feverish and somewhat nervous for a few days, and then menstruated as a girl at sixteen years of age. This she continued to do every month untll last July, when I was called to see her. Her nervous system was much affected during the period of menstruation; during the remainder of the time she appeared quite well. She had strabismus when sick.

I gave hyd. cum creta and rhei powders, and corrected the secretions and a slight constipation; then gave, two or three times each day, a small quantity of infusion of humulus. For the past year she has been perfectly well, and no signs of menstruation. The organs are normal. She is now six years of age.—*Chicago Med. Journal.*

